# Risk Factors for Retinopathy and DME in Type 2 Diabetes—Results from the German/Austrian DPV Database

**DOI:** 10.1371/journal.pone.0132492

**Published:** 2015-07-15

**Authors:** Hans-Peter Hammes, Reinhard Welp, Hans-Peter Kempe, Christian Wagner, Erhard Siegel, Reinhard W. Holl, DPV Initiative—German BMBF Competence Network Diabetes Mellitus

**Affiliations:** 1 5th Medical Department, University Medical Center, University of Heidelberg, Mannheim, Germany; 2 Department of Internal Medicine, Knappschafts-Krankenhaus, Bottrop, Germany; 3 Centre for Diabetes and Nutrition Ludwigshafen, Ludwigshafen, Germany; 4 Specialized Diabetes Practice, Saaldorf, Germany; 5 Department of Internal Medicine, St. Josefs Hospital, Heidelberg, Germany; 6 Institute of Epidemiology and Medical Biometry, University Medical Centre, Ulm, Germany; Eye Hospital, Charité, GERMANY

## Abstract

To assess the prevalence and risk factors for early and severe diabetic retinopathy and macular edema in a large cohort of patients with type 2 diabetes Retinopathy grading (any retinopathy, severe retinopathy, diabetic macular edema) and risk factors of 64784 were prospectively recorded between January 2000 and March 2013 and analyzed by Kaplan–Meier analysis and logistic regression. Retinopathy was present in 20.12% of subjects, maculopathy was found in 0.77%. HbA1c > 8%, microalbuminuria, hypertension, BMI > 35 kg/m^2^ and male sex were significantly associated with any retinopathy, while HbA1c and micro- and macroalbuminuria were the strongest risk predictors for severe retinopathy. Presence of macroalbuminuria increased the risk for DME by 177%. Retinopathy remains a significant clinical problem in patients with type 2 diabetes. Metabolic control and blood pressure are relevant factors amenable to treatment. Concomitant kidney disease identifies high risk patients and should be emphasized in interdisciplinary communication.

## Introduction

The eye is considered the primary target of chronic hyperglycemic damage in type 1 and type 2 diabetes [1]. Although a decreasing incidence of severe retinopathy has been observed during recent years in type 1 diabetes, the overall impact of diabetes on visual outcome appears to increase in type 2 diabetes [2]. Diabetic retinopathy (DR) is generally devided into incipient stages of vasoregression (early DR), and subsequent stages of responsive angiogenesis (severe DR) and/or of increased permeability (diabetic macular edema, DME) [3]. Both, early and severe stages in type 2 diabetes indwell specific risk factors which are amenable to intervention. In a recent meta-analysis of studies in which DR was firmly documented by retinal photographs, HbA1c and blood pressure were identified as modifiable risk factors, with diabetes duration and ethnicity as significant non-modifiable risk factor [4]. The prevalence of any retinopathy in type 2 diabetes was 25.2% and the prevalence of DME was 5.6%. The study also noted that the modifiable as well as the major non-modifiable risk factors applied broadly to the entire range of retinopathy stages. The relative contribution of dyslipidemia as a potential novel risk factor, however, was not assessed. Dyslipidemia and obesity are components of the metabolic syndrome which precede type 2 diabetes. Preceding overt diabetes, retinal endothelial dysfunction has been identified which ameliorates after bariatric surgery [5]. Therefore, obesity may serve as an independent risk factor for DR, but available data are controversial [6, 7].

Another modifiable risk factor for the development of DR in type 2 diabetes is smoking. Again, studies revealed conflicting results. In the Wisconsin Epidemiologic Study of Diabetic Retinopathy Study (WESDR), smoking was not a factor contributing to DR development, and in the United Kingdom Prospective Diabetes Study (UKPDS) smoking was even protective [8, 9].

As a third at least partially modifiable factor, diabetic kidney disease aggravates retinopathy progression. In type 1 diabetes, concomitant nephropathy in a patient with retinopathy is the strongest predictor for progression [10].Whether the concept of a renal-retinal syndrome can be extended to type 2 diabetes, is not clear.

Therefore, the aim of the present study was to identify the prevalence of diabetic retinopathy, the modifiable and non-modifiable risk factors in patients with type 2 diabetes, with a particular focus on parameters of the metabolic syndrome and on the diabetic kidney. We took advantage of a large, mostly Caucasian population from Germany and Austria reflecting current diabetes care according to identical guidelines and ascertained by comparable technology.

## Patients and Methods

### Ethics Statement

Analysis of anonymized routine data within the German/Austrian Diabetes Prospective Documentation Initiative (DPV) was approved by the Ethics Committee of the Medical Faculty of the University of Ulm, as reported earlier [11].

Methods of data collection including participating centers (see acknowledgment) have been published [12]. Details of the DPV software have been reported earlier [13]. Longitudinal anonymous patient records were accumulated from January 2000 until March 2013, using the DPV documentation and quality management system. Patients with type2 diabetes were included in the study when age at disease onset was above 40 years, and at least one retinal examination had been documented according to the guidelines of the German Diabetes Association [14]. In brief, type 2 diabetes was classified by specialised diabetologists(subspecialty degree of the German diabetes association or the GermanAssociation of Medical Doctors), based on German guidelines which areidentical to ADA and WHO guidelines (www.leitlinien.de/mdb/downloads/nvl/diabetes-mellitus/dm-therapie-1aufl-vers4-lang.pdf). Patients younger than 40 years of age were excluded, as differentiation from type-1 diabetes is more difficult in this age group. Patients with an onset of type-2 diabetes earlier than age 40 are a special subgroup, which differs from the typical type-2 patient with onset above 40 years of age [15, 16].

By March 2013, 195145 patients from 328 diabetes centres in Germany and Austria were continuously monitored. We excluded centres who reported lower than 50% retinopathy assessment rates leaving 85813 patients from 166 recruiting centers in Germany and Austria. Complete data sets on retinal examination and covariates were available from 64784 patients.

### Retinopathy

Screening and grading for the presence of retinopathy and DME was performed in accordance with published guidelines and details of the grading for retinopathy have been previously reported [12, 13]. Any retinopathy was recorded when at least mild non-proliferative diabetic retinopathy was observed in at least one eye. Severe retinopathy included severe non-proliferative diabetic retinopathy and proliferative diabetic retinopathy (the equivalent of ETDRS level ≥ 53). DME was defined as the presence of any retinal thickening or hard exsudates at the posterior pole irrespective of center involvement or ischemic characteristics. The rate of patients examined in the study exceeded 80%, and inter-rater agreement in selected centers approached a κ coefficient of 1.0. The age at the first pathological eye examination was defined as onset of retinopathy, and the worst eye defined the level of retinopathy.

### Covariates

We included the following independent risk factors into the analysis: age, diabetes duration, gender, HbA1c, hypertension, dyslipidemia and smoking (current and previous). All parameters were assessed according to established procedures.

### Statistical analysis

We used the SAS 9.3 statistics software package (SAS Institute, Cary, NC, USA) for data evaluation and analysis. This included descriptive analysis, Wilcoxon rank sum test for comparison of patient groups (without, with any, with severe retinopathy, with DME), survival analysis using Kaplan-Meier method to describe the presence of retinopathy in relation to covariate stratification, and logrank statistics for homogeneity testing among strata. Multiple logistic regression analysis was used to evaluate relative contributions of covariates (odds ratios and 95% CI) to the risk of early and severe retinopathy, and on macular edema. Bonferroni stepdown correlation of p-values (method of Holm) was used to adjust for multiple testing.

## Results

**Fig 1:**
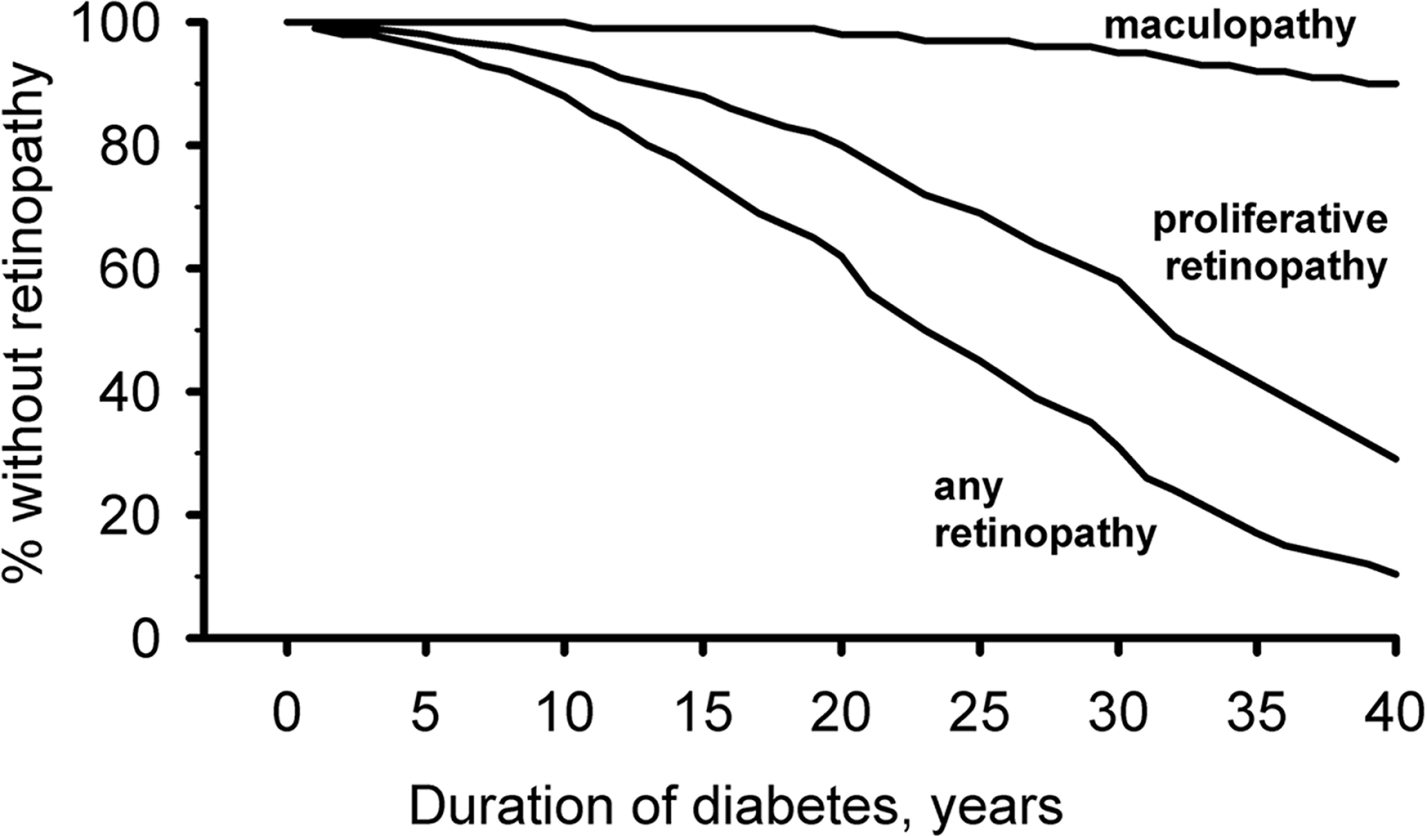
Kaplan-Meier analysis relating time to retinopathy (any versus severe versus DME) to duration of diabetes (individual survival curves are labeled).

Patient characteristics are summarized in Table 1. Retinopathy was absent in 79.88% of patients (51750) at the most recent visit. 10.26% (6646) had mild to moderate retinopathy, and 9.09% (5887) had severe non-proliferative or proliferative retinopathy. DME was reported in 501 patients (0.77%). Kaplan-Meier analysis revealed the presence of any retinopathy in 89,7%, of severe non-proliferative or proliferative retinopathy in 67.5% and of DME in 9,8% after 40 years (Fig 1). Table 1 shows significant differences between patients with and without retinopathy, and patients with DME. Compared with retinopathy-free patients, any and severe retinopathy were significantly associated with male gender, older age, longer diabetes duration, more frequent hypertension, worse diabetes control and dyslipidemia. The groups with severe eye lesions was proportionally more likely to receive antihypertensive and lipid lowering co-medication, in particular in the group with DME. We also analyzed differences between study patients and patients not included in the study because of missing eye examination. The latter group was modestly younger at diabetes discovery (delta 2.73 years), but had similar diabetes duration, slightly higher mean HbA1c (7.65 vs 7.36%), but lower proportions of hypertension (68.4 vs 76.3%), and reported modestly proportions of antihyperglycemic, antihypertensive, and antidyslipidemia therapies.

**Table 1:** Clinical and laboratory characteristics according to retinopathy status.

Characteristic	Patients by retinopathy status										P value^a^
	All patients		None		Mild		Severe		DME		
Patients (n)	64784		51750		6646		5887		501		
Male sex (%)	51.4		51.6		52.3		49.0		44.3		<0.0001
Age at last visit (years)	68.7	(0.04)	68.1	(0.05)	70.4	(0.11)	72.3	(0.12)	72.4	(0.41)	<0.0001
Diabetes duration (years)	9.2	(0.03)	8.1	(0.03)	13.9	(0.10)	13.6	(0.12)	15.6	(0.42)	<0.0001
Age at onset (years)	59.5	(0.04)	60.0	(0.05)	56.5	(0.12)	58.8	(0.15)	56.8	(0.50)	<0.0001
Insulin treatment (%)	44.5		39.9		60.9		63.4		82.6		<0.0001
Rapid-acting analogues (%)	28.3		26.8		32.9		31.8		26.2		
Long-acting analogues (%)	32.6		32.0		35.5		33.1		29.0		
Riskfactors											
Obese (%)	47.7		47.4		50.3		47.1		47.5		<0.0001
HbA1c (%)	6.1	(0.02)	6.0	(0.02)	6.3	(0.04)	6.8	(0.04)	7.5	(0.10)	<0.0001
BP systolic (mmHg)	135.5	(0.08)	134.9	(0.09)	138.0	(0.24)	137.5	(0.25)	139.1	(0.83)	<0.0001
BP diastolic (mmHg)	77.3	(0.05)	77.4	(0.05)	77.1	(0.14)	76.6	(0.14)	77.4	(0.43)	<0.0001
Total cholesterol (mg/dl)	198.1	(0.26)	199.2	(0.30)	195.7	(0.76)	193.4	(0.73)	194.7	(2.25)	<0.0001
LDL-cholesterol (mg/dl)	112.4	(0.23)	113.6	(0.27)	111.0	(0.72)	107.1	(0.63)	104.8	(2.11)	<0.0001
HDL-cholesterol (mg/dl)	49.4	(0.12)	49.4	(0.14)	49.0	(0.30)	50.1	(0.35)	47.5	(0.80)	NS
Triacylglycerol (mg/dl)	190.1	(0.63)	192.4	(0.73)	184.2	(1.84)	181.3	(1.70)	184.2	(4.96)	<0.0001
Smokers (cig/d)	1.49	(0.03)	1.59	(0.03)	1.16	(0.07)	1.06	(0.07)	1.51	(0.33)	<0.0001
Medication (%)^b^											
ACE inhibitors	26.7		25.8		30		29.7		37.7		<0.0001
Calcium antagonists	12.9		11.9		16.9		16.3		20.8		<0.0001
Diuretics	27.5		26.2		32.6		32.2		40.7		<0.0001
Beta blockers	25.2		24.9		27.1		25.6		32.9		<0.0001
ATR blockers (sartans)	9.9		9.4		11.5		11.8		12.4		<0.0001
Statins	20.6		20.3		22.8		20.7		19.6		NS
Fibrate	0.9		0.9		0.9		0.8		0.6		NS

Values represent mean (SE)

^a^ For p value comparison according to retinopathy status (four groups, non-parametric test, Wilcoxon's test, adjusted for multiple comparisons according to Holm)

^b^ Alone or in combination

Kaplan-Meier analysis revealed that after a diabetes duration of 20 years, 38.2% had any retinopathy, and after 40 years, the proportion had increased to 89.7%. The corresponding figures for severe eyes disease were 19,7% and 67,5%, and for DME 1,6 and 9,8%, respectively.

The cohort differed significantly in retinopathy-free survival when grouped for long-term HbA1c above or below a level of 7% (53mmol/mol) (25.3 vs 22.2 years, logrank test, chi-square 103.7; p<0.0001). Above this threshold for metabolic control, no significant difference in time to retinopathy was identified between groups.

We analyzed the effect of blood pressure on retinopathy onset using cutoffs between 130/80 and 140/90 mm Hg. There was a constant, highly significant difference in time to retinopathy over the entire range (for 140/80 mmHg 22.4 vs 24.7 years, logrank test, chi square 44.91; p<0.0001), suggesting the absence of any threshold below which retinopathy is being prevented.

BMI was analyzed for its potential to influence retinopathy development. We used the WHO cut-off of 30 kg/m^2^to identify a difference in time to retinopathy by univariate analysis and found a significantly higher risk of retinopathy in the obese subgroup (21.9 vs 24.6 years, chi square 79.54; p<0.0001).

Both, smoking (21.8 vs 23.1 years, chi square 24.79, p<0.0001) and male gender (22.44 vs 23.51, chi square 22.78; p<0001) were also significant contributors in univariate analysis.

As several of the above factors are interrelated (see Table 1), we used multivariable regression analysis to identify relevant independent contributors to any retinopathy. As shown in Table 2, male gender, HbA1c > 8%, hypertension (>140/80 mmHg), obesity (BMI > 35 kg/m^2^), and smoking, but not dyslipidemia were independently related to any retinopathy. When systolic and diastolic blood pressure were included, only diastolic pressure was significantly contributing.

**Table 2:** Multiple logistic regression analysis, any retinopathy.

Variable	OR	95% CI	P
Male gender	1,11	1,07–1,15	<0,0001
HbA_1c_>8%	1,34	1,29–1,39	<0,0001
RR>140/80 mm Hg	1,15	1,11–1,20	<0,0001
BMI >35 kg/m^2^	1,1	1,05–1,16	0,0005
Microalbuminuria	1,16	1,11–1,20	<0,0001

Nine percent of the entire cohort (5887 patients) had more severe retinopathy. Bivariate analysis revealed glycemic control, blood pressure, dyslipidemia, BMI, but not gender and smoking as relevant contributors. In the multivariable analysis, glycemic control (HbA1c threshold 8% (64 mmol/mol)), blood pressure (threshold 140/80 mm Hg), dyslipidemia, and smoking prevailed (Table 3). The analysis of maximum likelihood estimates revealed a significant albeit minor contribution of diastolic blood pressure to severe stages of retinopathy (HR 1.009, p < 0.0001), while systolic blood pressure was insignificant.

**Table 3:** Multiple regression analysis, severe retinopathy.

Variable	OR	95% CI	P
HbA_1c_>8%	1,21	1,137–1,279	<0,0001
RR>140/80 mm Hg	1,11	1,043–1,179	0,001
Microalbuminuria	1,2	1,14–1,271	<0,0001

DME was reported in 501 patients (0.77%). This subgroup had the highest age, the longest diabetes duration and the highest proportion with HbA1c > 7% (53 mmol/mol) and hypertension (RR>140/80 mmHg) despite the highest proportion receiving antihypertensive medication (65.9%, compared with 49.1% of the entire group) and insulin. Using the Wilcoxon test, age, diabetes duration, HbA1, and LDL-C, but not BMI, blood pressure and HDL-C, triglycerides and smoking were significantly associated with the development of DME, while gender, blood pressure (≥ 140/80 mm Hg) and the use of insulin were significant contributors in bivariable analysis after Bonferroni correction. Multiple regression analysis identified glycemic control, hypertension and dyslipidemia as the strongest covariates (Table 4).

**Table 4:** Multiple regression analysis, macula edema.

Variable	OR	95% CI	P
HbA_1c_>8%	1,57	1,288–1,903	<0,0001
RR>140/80 mm Hg	1,39	1,11–1,74	0,0041
Microalbuminuria	1,31	1,089–1,582	0,0042
Macroalbuminuria	2,77	2,105–3,655	<0,0001

Finally, the impact of diabetic nephropathy on the development of any retinopathy, severe retinopathy and DME was explored. Microalbuminuria increased the risk for retinopathy by 15.6% (95% CI 1.112–1.201; p<0.0001) and macroalbuminuria increased the risk by 28.4%(95% CI 1.118–1.388; p<0.0001). The presence of microalbuminuria on severe retinopathy raised the risk of retinopathy to 20.4%(95% CI 1.14–1.271; p<0.0001), and of macroalbuminuria raised the risk to50.5% (95% CI 1.36–1.673; p<0.0001).While microalbuminuria increased the risk for DME by 31.3% (95% CI 1.089–1.582; p<0.0001), macroalbuminuria resulted in an excess risk of 177% for DME (95% CI 2.105–3.655; p<0.0001).

## Discussion

Our study in a large cohort of subjects with type 2 diabetes revealed that retinopathy remains a significant microvascular complication with glycemia and blood pressure as the most important modifiable risk factors. As a novelty, our data identify macroalbuminuria as the strongest risk factor for DME. Obesity has a rather modest role in early, but not in severe retinopathy.

The significance of macroalbuminuria for vision threatening diabetic eye disease is clearly evident from our data. This is of particular clinical importance for two obvious reasons: a. a subgroup of patients exists with an extraordinary malignant clinical course, and b. this subgroup may not benefit from intravitreal before albuminuria is not improved by optimal medical care.

While the relative effect size of microalbuminuria on the three prespecified retinal phenotypes falls in between glycemia and blood pressure, macroalbuminuria exceeds this effect by at least twofold. The WESDR study did not find significant associations between early stages of kidney and retina lesions, but only when lesions were advanced (gross proteinuria and CSME) [10]. Higher statistical power of our study and group-specific differences may further explain discrepancies between results. In the cross-sectional part of the Jutland study, Knudsen et al. observed a significant association only for macroalbuminuria and CSME (OR 5.18) in a group of 328 type 2 diabetic subject which had higher glycemic levels (mean Hb1c 8.1%) and blood pressure (mean blood pressure 140/80 mm Hg)[17]. The smaller numbers of patients studied may have prevented finding a similar relation between DME and microalbuminuria as in the present study.

Mechanistically, vasoregression and increased capillary permeability are the two fundamental characteristics of early DR. Vascular hyperpermeability is a possible shared pathogenetic mechanism between the eye (DME) and the kidney (albuminuria). Knudsen et al. found that albuminuria and the rate of transcapillary albumin escape correlates with diabetic macular edema [18]. The defects underlying the breakdown of the blood-retinal-barrier are multiple, including elevated levels of permeability-enhancing growth factors, chronic hyperglycemia, overproduction of advanced glycation end products, and inflammation. The transition from the non-albuminuric to the albuminuric state in type 2 diabetes may be reflected by an increase in systemic inflammation, suggesting that the retinal phenotype is caused by systemic factors causing a retina-specific response[19]. Polypharmacy using antihyperglycemic, antihypertensive, antihyperlipidemic and antiplatelet drugs reduces retinopathy incidence in microalbuminuric type 2 diabetic patients, but the impact on DME remains to be studied [20].

We observed an effect of obesity on DR. This result is consistent with the Hoorn Study, however with a much smaller effect (odds ratio for retinopathy 3.52 for a BMI range between 28.4 and 45.7 kg/m^2^) [21]. Of note, the relationship between BMI and DR only exists in the diabetic vasoregression stage (any retinopathy), but not in more severe stages (i.e. increased permeability (DME) or angiogenesis (PDR)). Apart from adipokines which can influence arteriolar constriction, venolar dilatation, or both, resulting in a change in the arteriovenuous ratio (AVR), adipose tissue may submerge adipose derived stromal cells (ADSC) into the circulation which can modify vascular response to injury [22]. ADSCs are able to promote resistance to vasoregression in experimental diabetic retinopathy models [23], and can also provide proangiogenic potential, which would yield an either protective effect on DR along with increasing obesity, or a promotion of severe DR [22]. Neither was observed in our study which is consistent with the observation that ADSCs, when obtained from diabetic origin, may have reduced functionality. Functionality of ADSCs may also vary by age, as suggested by the TODAY study, in which obesity in adolescents was shown to protect from retinopathy [24].

Hyperglycemia, hypertension, and dyslipidemia are well defined modifiable risk factors, which are confirmed as such in our study. Given the size of the study population, calculated ORs reflect to a certain degree the strengths of the individual factors, glycemia being the strongest for any and severe retinopathy. This is in agreement with multiple previous studies, and also with a recent pooled analysis of the global prevalence of and risk factors for DR [4], suggesting that these risk factors apply broadly across all stages of DR. Also, blood pressure was found as a risk factor in the large metaanalysis and in our study, however with some differences [4]. The odds ratio of blood pressure on any retinopathy was moderate but became considerably higher with severe stages, in particular with DME. Hypertension, along with increases in vascular shear stress, overactivation of the renin-angiotensin system and overproduction of inflammatory cytokines cooperates with hyperglycemia in the generation of oxidative stress and inflammation [25]. Smoking contributes to this modifiable oxidative stress, and we provide robust data that smoking is detrimental and a risk factor, although the effect size is modest.

Some of the strengths of the study are also weaknesses. Ethnicity, sample size, consistency in phenotyping, and risk factor associations for severe retinopathy stages (DME, PDR) reduces heterogeneities usually present in other studies. The main weakness of the study is that diabetic retinopathy was not diagnosed by fundus photography, but by fundus examination by retinal specialists according to standardized protocols. Given the known kappa statistics for diagnosing DME by funduscopy as compared with fundus photography, we might envisage an underestimation of DME in our study [26]. However, recent evidence suggest that funduscopy by retinal specialists is in fair to moderate agreement with fundus photography in type 2 diabetes and has a very high specificity [27]. Our estimates of DME are lower than the estimates recently published on a global scale, but not different than those observed ten years ago in a large cohort in the north of Germany and in agreement with a recent analysis of the UK Health Improvement Network [28, 29]. Given the characteristics of a contemporary field study, it should be noted that the diagnostic procedure is the same that identifies patients with center-involving CSME. Furthermore, the numbers of patients identified with established DME is large enough to permit robust analysis of risk factors as the primary aim of the study.

We consider our study group being representative because of the only modest differences in relevant risk factor between the group which was included in the study and the group not included because of incomplete retinopathy reporting.

In conclusion, our study provides a large-scale estimate of DR and its current risk patterns in modern treatment settings. DR is less frequent than albuminuria in type 2 diabetic patients, and albuminuria is a strong risk factor for severe DR, in particular DME. Patients with type 2 diabetes and albuminuria should be carefully monitored for progressive eye disease, and patients with DME should be evaluated for concomitant kidney disease.
